# Do autistic adults spontaneously reason about belief? A detailed exploration of alternative explanations

**DOI:** 10.1098/rsos.231889

**Published:** 2024-07-31

**Authors:** Ruihan Wu, Jing Tian Lim, Zahra Ahmed, Rachael Berger, Ensar Acem, Ishita Chowdhury, Sarah J White

**Affiliations:** ^1^ Institute of Cognitive Neuroscience, University College London, London, UK; ^2^ Royal Free Hospital, NHS Foundation Trust, London, UK; ^3^ Department of Psychology, Kadir Has University, Istanbul, Turkey

**Keywords:** autism, spontaneous mentalizing, eye-tracking, false-belief

## Abstract

Southgate *et al.*’s (Southgate 2007 *Psychol. Sci.* 18, 587-92 (doi:10.1111/j.1467-9280.2007.01944.x)) anticipatory-looking paradigm has presented exciting yet inconclusive evidence surrounding spontaneous mentalizing in autism. The present study aimed to develop this paradigm to address alternative explanations for the lack of predictive eye movements on false-belief tasks by autistic adults. This was achieved through implementing a multi-trial design with matched true-belief conditions, and both high and low inhibitory demand false-belief conditions. We also sought to inspect if any group differences were related to group-specific patterns of attention on key events. Autistic adults were compared with non-autistic adults on this adapted implicit mentalizing task and an established explicit task. The two groups performed equally well in the explicit task; however, autistic adults did not show anticipatory-looking behaviour in the false-belief trials of the implicit task. Critically, both groups showed the same attentional distribution in the implicit task prior to action prediction, indicating that autistic adults process information from social cues in the same way as non-autistic adults, but this information is not then used to update mental representations. Our findings further document that many autistic people struggle to spontaneously mentalize others’ beliefs, and this non-verbal paradigm holds promise for use with a wide range of ages and abilities.

## 1. Introduction

### 1.1. Identifying autism and implicit mentalizing tasks

Identifying autism is not always easy. Some autistic people are not receiving a timely diagnosis and presumably others are not being identified at all. Indeed, adult diagnosis is increasingly common [1–4], autistic people with higher IQs are diagnosed later than those with lower IQs [5–7] and females, on average, receive their diagnosis considerably later than males and are more likely to have been previously misdiagnosed [2,8–11]. These have likely resulted in part from the current autism diagnostic framework, which still relies on clinicians’ interpretation of behaviours through observation and/or parental reports [12].

This diagnostic framework is problematic when considering compensation (or camouflaging). Although people who receive a diagnosis are likely to be least able to compensate or to have particular characteristics that cannot easily be camouflaged [12–15], this does not necessarily mean that the neurodevelopmental difficulties of those who can circumvent diagnosis have genuinely remitted at a cognitive level; rather, these difficulties persist, and such behavioural compensation comes at a great cost [12,13,16,17]. It is, therefore, critical to develop more sensitive assessments that are not susceptible to compensation and that target underlying cognitive ability [18].

Implicit mentalizing tasks hold promise as one such assessment. Southgate *et al.*'s [19] anticipatory-looking paradigm has been suggested to be able to detect more subtle false-belief reasoning than traditional explicit and even than some other implicit, mentalizing paradigms [19,20]. In this paradigm, participants were first familiarized with a puppet hiding an object in one of two boxes and an agent retrieving the object from the box by reaching through the corresponding window in an occluding screen between herself and the boxes. Next participants watched a single false-belief trial, in which the puppet moved the object from one box to the other and then removed it from the scene while the agent was not looking. Eye movements were recorded to assess which window they expected the agent to reach through. Southgate *et al*. found that non-autistic infants made eye movements toward the window/box that were consistent with the agent’s false belief about the object location (belief-congruent), indicating an ability to represent others’ false beliefs. However, a considerable number of infant studies have not replicated Southgate *et al.*'s [19] findings, and the authors now argue that this paradigm should not be used with infants [21]. In adults, evidence supports the idea that anticipatory paradigms can more reliably detect mentalizing (reviewed in Schneider *et al.* [22]), even though several adult studies have reported replication issues with this paradigm (reviewed in Poulin-Dubois *et al.* [23]). The potential challenges of this paradigm, which might lead to replication failures, and some suggested solutions are discussed in the following sections.

Senju *et al.* [24] provided the first evidence for a dissociation between implicit and explicit mentalizing task performance: autistic adults’ looking behaviour was not biased by the agent’s false belief, indicating that they were not spontaneously mentalizing, despite performing comparably to their non-autistic counterparts on explicit mentalizing tasks. Presumably, in the latter instance, autistic adults may ‘hack’ the solution through compensatory strategies, such as linguistic abilities or executive functions (e.g. [12,13,25–29]). These results have been replicated with autistic children (e.g. [30,31]) and adults (e.g. [32]). Yet, this promising finding has been challenged, as already mentioned, in terms of the reliability of the paradigm, but also in the interpretation of the data (e.g. [33–35]).

### 1.2. Challenges to implicit mentalizing tasks in autism research

Two specific challenges have been made about the reliability of the paradigms of Southgate *et al.* [19] and Senju *et al.* [24]. First, each participant was only presented with one test trial. This single-trial design is problematic because trial-by-trial variation is particularly large between participants, which can dramatically escalate error variance and the dropout rate; for example, 44% in Southgate *et al.* [19] infants, although only 3% in Senju *et al.* [24] adults and therefore attenuate reliability [33,36]. A multi-trial design would improve the signal-to-noise ratio and increase power, allowing for a better estimation of individual performance. Therefore, the current study first set out to increase the number of trials to improve task reliability.

Second, both studies only presented a false-belief condition; no matched true-belief condition, in which the observer’s belief should be consistent with that of the agent, was included as a baseline. This lack of control condition opens the door to alternative explanations. One prominent example is by Heyes [35], who proposed that non-autistic people pass this task owing to submentalizing rather than mentalizing. Specifically, the submentalizing hypothesis claims that non-autistic people exhibit belief-congruent anticipatory looking in false-belief trials because they get distracted by the agent’s head turning and, therefore, do not pay attention to or remember the subsequent object displacement. Heyes [35], therefore, argued that non-autistic people predict the agent’s action based on their own false belief of the object’s location, rather than the agent’s false belief. Additionally, she claimed that autistic people are less distracted by the agent and hence know the object is not in either box but are simply less likely to predict people’s actions.

Accordingly, poor performance on the commonly reported outcome measures of this task alone cannot be used to conclusively deduce that autistic people have difficulties in spontaneous mentalizing. Thus, the second aim of the current study was to address this by including true-belief conditions that closely match false-belief conditions and providing a detailed analysis of eye movements throughout the paradigm. Based on Heyes's [35] submentalizing hypothesis, we should see differences between the participant groups in visual attention to the key events. Specifically, at the onset of the head-turn period in both false-belief and true-belief conditions, non-autistic people should attend and, therefore, fixate more on the agent but less on the puppet moving the object than autistic people. Also, autistic people should be less likely to predict the agent’s action in both false-belief and true-belief trials.

### 1.3. Overcoming these challenges and the current study

Several studies have endeavoured to improve the reliability of Southgate *et al.*'s [19] paradigm. However, the results from studies implementing true-belief conditions are mixed. For example, non-autistic infants and adults were observed to be able to attribute both true beliefs and false beliefs with low cognitive demands [37,38]; but, with the same paradigm, Kulke *et al*. [39] did not find a relationship between the two in any age groups. Using the same task as Senju *et al.* [24], Gliga *et al.* [40] considered one of the familiarization trials as a true-belief condition and concluded that siblings of autistic children were able to attribute the agent’s true belief, but not false belief. However, this familiarization trial was shorter and simpler than the false-belief condition and did not involve a head turn. Also, as the agent’s true belief was consistent with reality and the child’s own belief, plus the agent’s reaching action was presented in every familiarization trial, it is possible that they predicted the agent’s action according to their own belief [38,41,42] or learned the behavioural contingency from the repeated action [43,44]. Therefore, it must be verified whether autistic people struggle specifically with representing false beliefs with a well-matched true-belief condition.

To date, the empirical results from studies adopting a multi-trial design are also mixed. Schneider *et al.* [45] were the first to investigate how spontaneous mentalizing operates over time in non-autistic adults and claimed that spontaneous mentalizing can be sustained over the course of a multi-trial procedure. Using the same paradigm in autistic adults, Schneider *et al.* [32] replicated the observations of Senju *et al.* [24]: autistic people did not spontaneously mentalize across the trials, despite performing well in explicit mentalizing tasks, indicating that multi-trial designs are viable and are not susceptible to compensatory learning in autistic people.

However, it is important to note that Schneider’s studies did not analyse whether participants showed a clear-looking bias towards the belief-congruent box. Instead, they compared the looking bias towards the belief-congruent box on false-belief trials and the belief-incongruent box on true-belief trials. Thus, it is unclear whether the belief manipulation was successful within each condition; indeed, the autistic participants surprisingly appeared similarly likely to look at either box in the true-belief condition [32, Figs. 2 and 3], and the non-autistic participants appeared more likely to look at the belief-incongruent location in the false-belief condition [45, Figs. 2 and 3]. Additionally, Schneider’s studies consisted of 20 test trials, each more than 1 min in duration, plus at least 20 familiarization trials, amplifying the total duration of the task. The present study, therefore, chose to make the paradigm more streamlined by removing any unnecessary actions and potential social confounds (i.e. an extra object displacement in the original false-belief trials and the agent’s wave and smile) and keeping familiarization trials to a minimum.

Another factor worth highlighting in Schneider *et al.* [32] is that, during the anticipatory period, both groups allocated their first fixation to the agent’s face in more than 70% of trials. This meant that more than 70% of their data was excluded from the analysis. One possible reason could be the absence of an occluder between the agent and the scene, a disparity with Southgate *et al.*'s [19] paradigm. It is possible that by removing the occluder, participants looked to the agent in anticipation of her action rather than making anticipatory saccades to the belief-congruent area as the first place where the action was expected. A second reason may be that the agent left the room, rather than turning to the back as in Southgate *et al.* [19]. This meant that the participant could not be sure of the agent’s knowledge of the object’s location while off-scene. A further contention is whether the reappearance of the agent is a salient event, which could result in retroactive memory interference on object displacement during the agent’s absence [35]. Moreover, it is worth noting that in Schneider *et al.* [32], the object was displaced twice in the true-belief scenario but only once in the false-belief scenario before the agent came back. Therefore, the higher memory load required in the true-belief condition may have caused the lack of looking difference between the belief-congruent area in true-belief trials and the belief-incongruent area in false-belief trials in both autism and non-autism groups. In order to avoid these potential caveats and, by doing so, increase the number of trials included in the analysis, we chose to retain the occluder, keep the agent visible at all times and displace the object only once.

Furthermore, both of Schneider’s studies used a false-belief condition with high-inhibitory demands, as the object was displaced to the other box, rather than removed from the scene (i.e. low-demand) as in Southgate *et al.* [19]. Wang and Leslie [38] directly contrasted high- and low-demand false-belief conditions. They found that both non-autistic 3-year-olds and adults showed clear anticipatory-looking behaviours towards the belief-congruent area in the low-demand condition, but no looking bias in the high-demand condition. As a result, they suggested that the high-demand scenario requires greater cognitive resources to inhibit one’s own belief about the object’s location. This same suggestion of a reality bias (or true-/own-belief bias) has also been attributed to autistic people as an explanation for their poor false-belief task performance compared to non-autistic people [41,42]. If this inhibition difficulty is indeed even stronger in autistic people, they may therefore, show a bias to look towards the object’s current location in the high-demand false-belief condition, rather than the lack of bias shown by non-autistic people. Accordingly, we chose to study the effectiveness of our belief manipulation and compare high- and low-demand false-belief conditions in both autistic and non-autistic people.

Schuwerk *et al.* [43] also reported a multi-trial study across just two trials, suggesting that experience might improve autistic people’s performance when the outcome action (i.e. the agent opening the belief-congruent window and retrieving the object) is shown; only the second trial tested this learning effect. Autistic and non-autistic adults differed in looking bias in the first false-belief trial, consistent with Senju *et al.* [24], but not in the second. However, the looking bias of autistic adults did not differ from chance in either trial, and no improvement was seen in autistic children [31]. Moreover, any improvement in performance by the autism group could be owing to compensatory learning of a behavioural contingency during the first trial, without representing the agent’s mental state [44]. Hence, we chose not to show the outcome action in experimental trials. If the improved performance observed by Schuwerk *et al.* [43] was owing to an increase in mentalizing, then the performance of autistic people in our task should also increase over time to the level of non-autistic people. On the other hand, in line with Schneider *et al.* [32], we expect to see no change in performance over time.

To summarize, the existing adaptations to the original Southgate *et al.* [19] paradigm have presented exciting, yet inconclusive, evidence surrounding spontaneous mentalizing in autism. The present study, therefore, sought to advance the paradigm by implementing a multi-trial experiment with shorter trials, matched true-belief conditions and both high- and low-inhibitory demand false-belief conditions to scrutinize the claims that have been made in the literature. In the low-demand false-belief condition, according to the literature [24], we predicted that autistic people should show no looking bias, while non-autistic people should be able to anticipate the agent’s false-belief-based action. In the high-demand false-belief condition, we expected both groups to show no looking bias [38]; this condition was included to address alternative explanations, in particular the submentalizing hypothesis and the reality bias hypothesis, rather than for assessing spontaneous mentalizing in our adapted implicit mentalizing task. In the true-belief conditions, we predicted both groups would look significantly longer at the belief-congruent than the belief-incongruent area [41]. Following Gliga *et al.* [40], we also expected both groups to show belief-congruent performance in the familiarization trials. Also, we expected attentional bias differences between the groups during the object displacement [35]. In addition, our second aim was to replicate Senju *et al.* [24] finding regarding the dissociation in performance between implicit and explicit mentalizing tasks. To prevent a potential ceiling effect in the simple explicit task used by Senju *et al.* [24], we selected a more advanced mentalizing task and predicted that autistic adults would perform similarly to non-autistic adults in this explicit mentalizing task, despite differences in the implicit task.

## 2. Material and methods

### 2.1. Participants

Participants were recruited through local participant databases, local autism support groups and advertisements placed around the local community. This study was approved by the local Research Ethics Committee, and all methods were performed in accordance with the approved guidelines and regulations. A written informed consent was obtained from all participants. Assuming a medium effect size (*F* = 0.25) as seen in studies by Senju *et al.* [24] and Schneider *et al.* [32] and power of 0.80, a sample size calculation indicated that we needed 17 participants per group to detect the critical interaction between group and belief. A total of 67 participants were recruited, 40 before and 27 after the COVID−19 pandemic. Participants were over-recruited to allow for the potentially high drop-out rate seen in some previous studies so that we might ensure a close match for age and non-verbal reasoning between the groups. Five participants (three autistic and two non-autistic) were excluded from the analysis owing to poor data quality (see *Data pre-processing* below), leaving 32 autistic adults, aged 18–64 years; and 30 non-autistic adults, aged 18–50 years. The resulting two groups were comparable for age, sex, verbal IQ, performance IQ and and full-scale IQ, as measured by the Wechsler Abbreviated Scale of Intelligence, Second Edition (WASI-II; [46]) and all participants had a full-scale IQ greater than 80 (table 1).

**Table 1.** Descriptive statistics of each group, mean (s.d.) and group-wise comparison.

	autism (*n* = 32)	non-autism (*n* = 30)	autism vs non-autism
age	32.00 (13.84)	30.70 (10.41)	*t*(60) = 0.42, *p* = .679, *d* = 0.106, BF_01_ = 4.822
sex (M : F)	17 : 15	16 : 14	χ^2^(1) = 0.99, *p* = .594, odds ratio = 1.008
verbal IQ (WASI-II)	116.29 (17.17)	115.77 (18.06)	*t*(59) = 0.12, *p* = .908, *d* = 0.030, BF_01_ = 5.150
performance IQ (WASI-II)	117.65 (21.09)	117.93 (18.83)	*t*(59) = −0.06, *p* = .955, *d* = −0.014, BF_01_ = 5.175
full-scale IQ (WASI-II)	121.37 (21.14)	120.33 (18.12)	*t*(59) = 0.21, *p* = .838, *d* = 0.053, BF_01_ = 5.083
autistic traits (AQ)	33.55 (7.65)	17.47 (7.36)	*t*(59) = 8.37, *p* < .001, *d* = 2.143, BF_01_ < 0.001
ADOS−2	7.81 (3.87)		

ADOS-2, Autism Diagnostic Observation Schedule Second Edition (data was unavailable for 16 autistic participants); AQ, Autism-Spectrum Quotient; WASI-II, Wechsler Abbreviated Scale of Intelligence, Second Edition.

None of the non-autistic participants reported a diagnosis or family history of psychiatric or neurodevelopmental conditions. Each participant in the autism group had previously received a diagnosis of autism spectrum disorder, Asperger syndrome, high-functioning autism or atypical autism from a qualified clinician. The Autism Diagnostic Observation Schedule Second Edition (ADOS-2 [47]) was available to verify the autism diagnosis for 16 autistic participants (table 1); nine of those met the criteria for autism or autism spectrum classification. The ADOS-2 score was not available for 16 autistic participants owing to test session logistics or participants’ personal decision to not take part in it. The seven participants who scored below the threshold were retained within the sample because scores below the cut-off are not uncommon in highly intelligent adults [48], and the autism group also reported significantly higher autistic traits on the AQ than the non-autism group (table 1). Furthermore, the results remained largely the same when we excluded those seven participants (see electronic supplementary material).

### 2.2. Procedure

Participants started the session by completing the WASI-II, then Section A of the implicit mentalizing task, followed by the explicit mentalizing task and questionnaires measuring demographics and autistic traits, and finished with Section B of the implicit mentalizing task. Participants were then fully debriefed. The overall duration of the experiment was 1.5 h.

### 2.3. Mentalizing tasks

#### 2.3.1. Implicit mentalizing task

**Figure 1.**
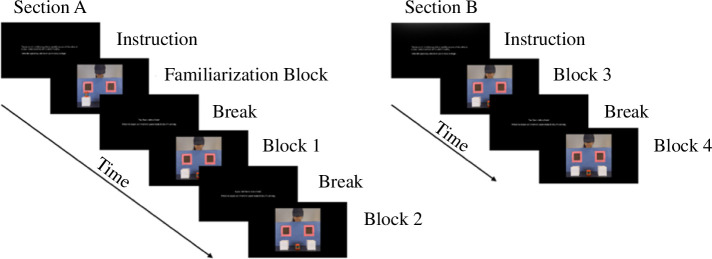
Implicit mentalizing task procedure.

The implicit mentalizing task used a multi-trial anticipatory-looking paradigm with matched true-belief and false-belief conditions, which was adapted from the anticipatory-looking paradigm in Southgate *et al.* [19]. The task was divided into segments (figure 1) with a 20-min break in between in an effort to keep participants’ attention. Participants were instructed to passively view some videos and informed they would be asked questions about their content at the end, to encourage them to pay attention and watch carefully. The questions asked about basic features of the videos (e.g. the colour of the puppet) and participants’ judgements (e.g. the most frequent final location of the object), but participants were not informed of the style of question in advance to avoid directing their attention to particular features of the videos.

**Figure 2.**
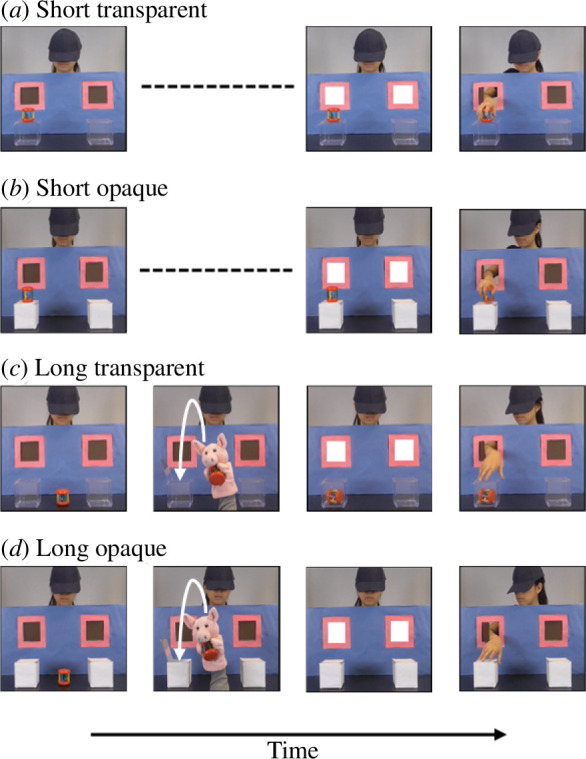
Short (5000 ms) and long (15 000 ms), opaque and transparent familiarization trials. The scenarios of (*a*) and (*c*) were identical to (*b*) and (*d*), respectively. Long familiarization scenarios include an additional object transfer event.

Section A contained one familiarization block, and both Sections A and B contained two experimental blocks (figure 1). The familiarization block included four short and four long familiarization trials, which enabled participants to learn the contingency that the agent would retrieve the object after an alert signal (the windows illuminated and a chime sounded simultaneously for 800 ms). The short familiarization trials started with the object on top of one of two boxes (figure 2*b*). The scene was frozen for 2800 ms from the onset of the alert signal. The agent then reached through the corresponding window and retrieved the object. During the long familiarization trial, a puppet hid the object in one of the boxes while the agent was watching (figure 2*d*). After the puppet left the scene, the alert signal occurred and the scene froze; then, the agent reached through the window, opened the box and retrieved the object. To make the contingency between the alert signal and the agent reaching through the window more salient, we also filmed two short and two long familiarization trials using transparent boxes to give participants a more direct perception of the object location (figure 2*a*,*c*). The end location of the object was counterbalanced in the short and long, transparent and opaque trials and producing eight possible videos, which were all displayed in the familiarization block in random order.

**Figure 3.**
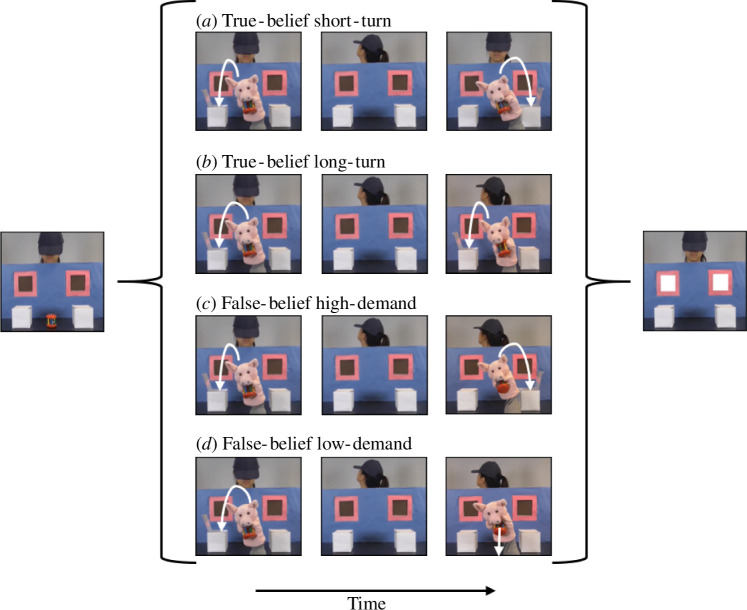
Sequence of events in the true-belief and false-belief condition videos (*a*) true-belief short-turn (37 000 ms), (*b*) true-belief long-turn (33 000 ms), (*c*) false-belief high-demand (37 000 ms) and (*d*) false-belief low-demand (33 000 ms).

Each experimental block started with one short and one long familiarization trial, randomly selected from the eight videos without replacement, to remind participants of the contingency. This was followed by four true-belief and four false-belief trials, consisting of two true- and two false-belief conditions: true-belief short-turn, true-belief long-turn, false-belief high-demand and false-belief low-demand. True-belief short-turn and true-belief long-turn conditions were matched to false-belief high-demand and false-belief low-demand conditions, respectively. In the true-belief conditions, the agent’s belief about the object’s location was congruent with its actual location, while these two locations were incongruent in the false-belief conditions so the agent held a false belief about the object’s location. These conditions only used the opaque boxes and the agent did not retrieve the object; instead, the scene remained frozen for the full 4800 ms from the onset of the alert signal (figure 3).

In the true-belief short-turn condition (figure 3*a*), the puppet hid the object in one of the boxes. A doorbell then rang and the agent turned away from the scene, followed almost immediately by the sound of a door closing, whereupon the agent turned back to the scene and witnessed the puppet move the object to the other box. Once the puppet had disappeared, the alert signal occurred and the scene froze. In the true-belief long-turn condition (figure 3*b*), the only difference was that the puppet returned the object to the original box while the agent was turned away from the scene and the agent did not turn back until the puppet had disappeared, at which point the alert signal occurred. The false-belief high-demand condition (figure 3*c*) only differed from the true-belief short-turn condition in that the agent remained turned to the back while the puppet moved the object to the other box. The false-belief low-demand condition (figure 3*d*) was similar to the true-belief long-turn condition except the puppet removed the object from the scene while the agent was turned away. Each sound was paired with the same corresponding event in all of the experimental videos, and the agent’s head movements always followed the puppet’s movement when she was facing the front to indicate that she was paying attention to the situation.

The box that first contained the object and the direction in which the agent turned were both counterbalanced, producing four possible videos for each condition. In each experimental block, two videos were randomly selected from each condition, giving a total of eight videos presented in random order. Participants watched each experimental video once in each section. Mathematica (Wolfram Research, United States, Inc. version 11.1) was used to code the random presentation sequences of the videos, which were then imported into the presentation software.

##### 2.3.1.1. Apparatus

A remote screen-based Tobii Pro X3−120 eye-tracker system, with a sampling rate at 120 Hz, was used to record gaze data (Tobii, Sweden). Visual and auditory stimuli were presented via a Dell Precision 5520 laptop (15.6-inch) with Tobii Pro Studio 3.4.8 software, integrated with the eye-tracker. Participants sat approximately 70 cm from the eye-tracker and were instructed to sit still throughout the eye-tracking assessment. A 9-point calibration was performed before each section began.

##### 2.3.1.2. Areas of interest (AOIs)

**Figure 4.**
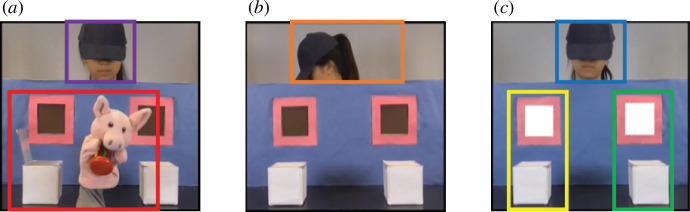
Examples of the areas of interest: (*a*) *Head_1* and *Head_3* (purple), *Puppet_1* and *Puppet_3* (red); (*b*) *HeadTurn_2* and *HeadTurn_4* (orange); (*c*) *Head_af* (blue), *Belief-congruent* (yellow) and *Belief-incongruent* (green).

Nine AOIs within five timeframes were identified across each trial (table 2 and figure 4). The total fixation duration was encoded and extracted through Tobii Pro Studio, measuring the sum of the duration of all fixations within each AOI. According to the scenarios, timeframes *1* and *3* captured object displacement, timeframes *2* and *4* captured the agent’s head-turn and timeframe *5* (*af*) captured action anticipation after the onset of the alert signal. Therefore, to investigate group differences in attention distribution, the total fixation durations of *Head_1* and *Head_3* were combined as *Head_bf*, *Puppet_1* and *Puppet_3* were combined as *Puppet_bf*, *HeadTurn_2* and *HeadTurn_4* were combined as *HeadTurn* and that of *Belief-congruent* and *Belief-incongruent* were combined as *Anticipation_af*. For the long familiarization trials using opaque boxes, the total fixation durations of *Belief-congruent* and *Belief-incongruent* for two different timeframes were extracted (table 2). For timeframe *5,* 4800 ms AOIs were used to evaluate if participants were able to predict the agent’s action by mentalizing about her beliefs, while 2500 ms AOIs were used in familiarization trials to examine if they paid attention and learned the contingency of the task.

**Table 2.** Definition of each AOI*.*

*AOI*_*timeframe*	location	event
*Head_1*	agent’s head area	the agent watches the puppet hiding the object in one of the boxes
*Puppet_1*	puppet’s moving area	
*HeadTurn_2*	agent’s head area	the agent turns away from the scene
*Head_3*	agent’s head area	the puppet displaces the object
*Puppet_3*	puppet’s moving area	
*HeadTurn_4*	agent’s head area	the agent turns back to the scene
*Head_af*	agent’s head area	from the onset of the alert signal to the end of the trial, total duration of 4800 ms; for the long familiarization trials using opaque boxes, data were also encoded from the onset of the alert signal up until the agent reaches through the window, total duration 2500 ms
*Belief-congruent*	window & box area consistent with agent’s belief	
*Belief-incongruent*	window & box area inconsistent with agent’s belief	

##### 2.3.1.3. Data pre-processing

Differential looking scores (DLS), which measure participants’ looking preference between two visual targets, were calculated by dividing the difference between the total looking time to the *Belief-congruent* and *Belief-incongruent* AOIs by the sum of the two. DLS ranged from 1 to −1, closer to 1 if participants showed a looking bias towards the *Belief-congruent* AOI, closer to −1 if they were biased towards the *Belief-incongruent* AOI and closer to 0 if they looked equally to both AOIs, equivalent to chance performance.

Three exclusion criteria were applied to ensure participants were paying attention to the key events in the videos (e.g. watching the hand retrieve the object). First, the data from the entire task were excluded for any participant whose average DLS in the familiarization block (based on the full 4 800 ms post-flash) was missing or below a chance to confirm that they had paid attention to the key event (a combination of the prediction and the action itself). Second, the data from each experimental block were excluded if the average DLS of the two familiarization trials at the beginning of that block was missing or below chance. Third, participants were excluded if they missed more than 25% of the data. After data cleaning, we excluded five participants (three autistic and two non-autistic), and removed the data from one experimental block for two additional participants.

### 2.3.2. Explicit mentalizing task

As our participants were adults and had average-to-high IQs, simple explicit tests to assess mentalizing ability tend to suffer from ceiling effects. Thus, the Strange Stories Task [49,50], an advanced mentalizing test, was used to assess participants’ ability to infer mental states in social situations explicitly, which was deemed suitable for the present cohort. To avoid a lengthy test session, only the mental state set of stories was used in the current study. In addition to accuracy (maximum score of 16), comprehension time was recorded (i.e. time elapsed from the start of reading a story to the start of answering the question).

### 2.4. Autistic traits

Autistic traits were measured by the Autism-Spectrum Quotient (AQ; [51]), with higher scores indicating more autistic traits, ranging between 0 and 50.

## 3. Results

All the data were analysed using IBM SPSS Statistics (version 29). Both frequential and Bayesian approaches were used and reported. Independent and repeated samples normal Bayesian analyses were used as an alternative to mixed-design analysis of variance (ANOVA) in the frequential approach. We used a diffuse prior to assuming unequal variance for two reasons: first, the prior on variance for our adapted implicit mentalizing task was unknown for both groups and second, we wanted the data analysis to be more objective and less influenced by subjectively elicited priors. The BF_01_ was reported (i.e. evidence for the null hypothesis divided by evidence for the alternative hypothesis). Thus, lower values indicate stronger evidence for the alternative hypothesis (e.g. <0.010 extreme evidence, 0.033–0.100 strong evidence, 0.100–0.333 moderate evidence and >1 no evidence).

### 3.1. Implicit mentalizing task

#### 3.1.1. Differential looking scores (DLS)

One-sample *t*-tests, comparing the average DLS of the long familiarization trials with opaque boxes (based on the first 2,500 ms post-flash, before the agent reached through the window) to chance performance, were conducted in each group separately to assess action prediction. Both groups performed significantly above chance: autism: *M* = 0.27, s.d. = 0.36, *t*(31) = 4.17, *p *< .001, *d* = 0.737, BF_01_ = 0.009; non-autism: *M* = 0.26, s.d. = 0.44, *t*(29) = 3.23, *p* = .003, *d* = 0.590, BF_01_ = 0.092 and no difference was found between the groups, *t*(60) = 0.07, *p* = 0.943, *d* = 0.018, BF_01_ = 5.206, indicating that both groups were able to correctly predict the agent’s actions.

The same tests were conducted for each experimental condition in the non-autism group to check the task validity. The DLSs for the true-belief short-turn (*M* = 0.22, s.d. = 0.42), *t*(29) = 2.79, *p* = .009, *d* = 0.510, BF_01_ = 0.245, true-belief long-turn (*M* = 0.32, s.d. = 0.36), *t*(29) = 4.91, *p*<.001, *d* = 0.897, BF_01_ = 0.001 and false-belief low-demand conditions (*M* = 0.19, s.d. = 0.34), *t*(29) = 3.00, *p* = .005, *d* = 0.548, BF_01_ = 0.115, were significantly above chance, but that of the false-belief high-demand (*M* = −0.10, s.d. = 0.30) did not significantly differ from zero, *t*(29) = −1.90, *p* = .068, *d* = −0.346, BF_01_ = 1.362. That is, non-autistic participants showed a preference for the *Belief-congruent* location in the true-belief short-turn, true-belief long-turn and false-belief low-demand conditions, but did not show this same looking bias in the false-belief high-demand condition. This indicated that all the conditions except the false-belief high-demand were valid for detecting implicit mentalizing ability.

Regarding the performance of the autism group on each condition, the results revealed that the DLS was significantly above zero in true-belief but not false-belief conditions: true-belief short-turn (*M* = 0.18, s.d. = 0.39), *t*(31) = 2.62, *p* = 0.014, *d* = 0.462, BF_01_ = 0.359, true-belief long-turn (*M* = 0.14, s.d. = 0.33), *t*(31) = 2.49, *p* = 0.018, *d* = 0.440, BF_01_ = 0.464, false-belief low-demand (*M* = 0.07, s.d. = 0.37), *t*(31) = 1.05, *p* = 0.304, *d* = 0.185, BF_01_ = 4.321, false-belief high-demand (*M* = −0.06, s.d. = 0.28), *t*(31) = −1.14, *p* = 0.264, *d* = −0.201, BF_01_ = 3.928. Since the false-belief high-demand condition was not valid, we decided to focus the following analysis on the false-belief low-demand condition and its matched true-belief long-turn condition.

**Figure 5.**
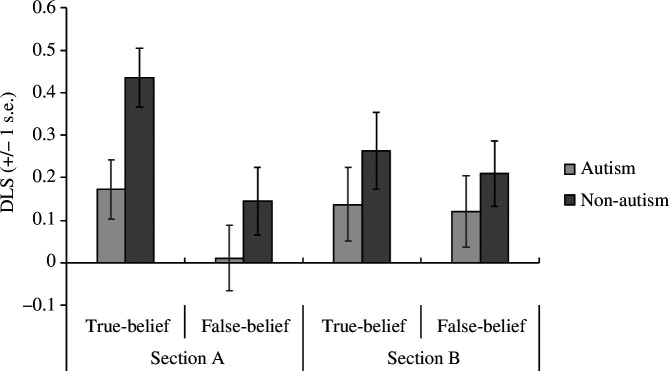
Mean true-belief and false-belief DLS of the implicit mentalizing task in Sections A and B for both the autism and non-autism groups.

**Figure 6.**
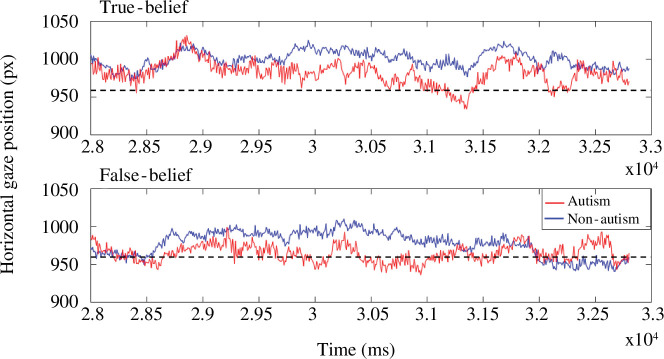
Grand averaged horizontal gaze position in response to stimulus presentations across true-belief and false-belief conditions in the two groups. This timeframe begins at the onset of the alert signal until the end of the video, from 2.8 to 3.28 × 10^4^ ms. The origin (0 px) of the frame is defined as the edge of the screen on the belief-incongruent side. The dashed line indicates the midline of the screen. Values above the dashed line are, therefore, biased towards the *Belief-congruent* side of the screen.

A 2 × 2 × 2 mixed-design ANOVA was conducted using the DLS as the outcome variable, with Time (Section A versus Section B) and Belief (true-belief versus false-belief) as within-subject factors and Group (non-autism versus autism) as a between-subject factor. There was a marginal main effect of Group, *F*(1, 60) = 3.88, *p* = 0.054, partial *η^2^* = 0.061, BF_01_ = 0.928, with the non-autism group displaying higher DLSs than the autism group (figures 5 and 6). The results also revealed a main effect of Belief, *F*(1, 60) = 7.67, *p* = 0.007, partial *η^2^* = 0.113, BF_01_ = 0.300; the means indicate that higher DLSs occurred in the true-belief than false-belief condition (figures 5 and 6). There was also a Time by Belief interaction, *F*(1, 60) = 4.82, *p* = 0.032, partial *η^2^* = 0.074, BF_01_ = 1.023 (figure 5). Post hoc tests (with α-level adjusted to *p* = 0.0125 for multi-comparison correction) indicated that the true-belief condition showed more of a belief-congruent bias than the false-belief condition in Section A, *t*(61) = 3.56, *p *< .001, *d* = 0.453, BF_01_ = 0.034, but not Section B, *t*(61) = 0.53, *p* = 0.597, *d* = 0.068, BF_01_ = 8.722. No other main effects or interactions were significant.

#### 3.1.2. Fixation Pattern

**Figure 7.**
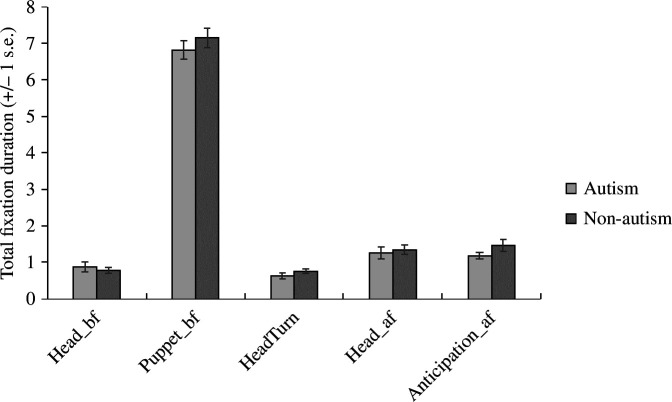
The mean total fixation duration indicated no group difference during all key events.

**Figure 8.**
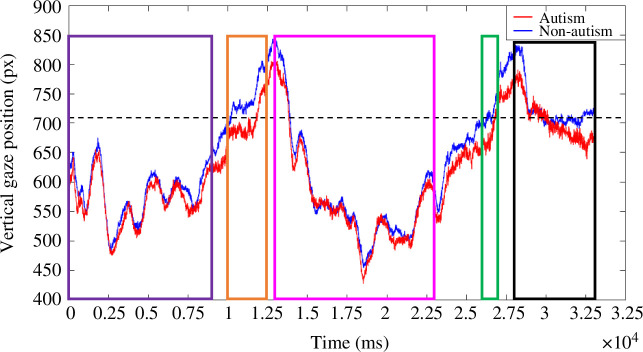
Grand averaged vertical gaze position in response to stimulus presentations across true-belief and false-belief conditions. Timeframe *1: Head_1* and *Puppet_1* (purple), Timeframe *2: HeadTurn_2* (orange), Timeframe *3: Head_3* and *Puppet_3* (pink), Timeframe *4: HeadTurn_4* (green) and Timeframe *5: Head_af* and *Anticipation_af* (black). Video onset occurred at 0 ms. The origin (0,0) of the frame is in the bottom left corner of the screen. The dashed line indicates the edge of the purple board in the scene.

We explored group differences in fixation patterns on the total fixation durations at critical time points. Given the critical frames were identical in all true-belief and false-belief trials and we found no interaction between group and belief, the data were collapsed across these two conditions. No group difference was found in *Head_bf*, *t*(60) = 0.54, *p* = 0.590, *d* = 0.138, BF_01_ = 4.564, *Puppet_bf*, *t*(60) = −0.91, *p* = 0.368, d = −0.231, BF_01_ = 3.589 or *HeadTurn*, *t*(60) = −1.19, *p* = 0.237, *d* = −0.304, BF_01_ = 2.735 (figure 7). More specifically, participants looked significantly longer at *Puppet_3* (*M* = 7.37, s.d. = 1.59) than *Head_3* (*M* = 0.88, s.d. = 0.74) while the agent was turned away, *t*(61) = 27.53, *p* < 0.001, *d* = 3.50, BF_01 _< 0.001 and this result held within each group (autism: *p* < .001, BF_01 _< 0.001; non-autism: *p* < 0.001, *BF*_*01* _< 0.001). Indeed, as shown in figure 8, the gaze patterns of both groups were almost identical in timeframes 1, 3 and 4. During timeframe 2, non-autistic adults seemed to mostly look at the agent (i.e. above the dashed line, indicating the top of the occluder), while autistic adults gazed at both the agent and the scene. Autistic participants seem, on average, to spend most of the timeframe 5 gazing within the belief-congruent and -incongruent areas (i.e. below the dashed line), whereas non-autistic participants gazed at both the agent and the anticipatory areas. However, there was no group difference in *Head_af*, *t*(60) = −0.45, *p* = 0.655, *d* = −0.114, BF_01_ = 4.759, or *Anticipation_af*, *t*(60) = −1.49, *p* = 0.141, *d* = −0.379, BF_01_ = 1.914. Altogether, these analyses indicated that the looking pattern of the two groups did not significantly differ during the key events.

### 3.2. Explicit mentalizing task

Independent samples *t*-tests revealed that performance on the Strange Stories Task was comparable between the autism and non-autism groups, both in terms of accuracy (autism: *M* = 13.31, s.d. = 1.93; non-autism: *M* = 13.63, s.d. = 2.28), *t*(60) = −0.60, *p* = 0.551, *d* = −0.152, BF_01_ = 4.428 and comprehension time (autism: *M* = 28.93, s.d. = 10.55; non-autism: *M* = 27.00, s.d. = 6.74), *t*(57) = 0.82, *p* = 0.417, *d* = 0.214, BF_01_ = 3.759.

## 4. Discussion

The present study aimed to probe spontaneous mentalizing in autism. To overcome the methodological difficulties seen in previous work, a multi-trial paradigm with well-matched true-belief control conditions was used. We conducted a detailed analysis of gaze patterns throughout individual trials, as well as changes in performance over the test session. Our results, to an extent, support the presence of spontaneous mentalizing difficulties in autistic adults despite the typical allocation of attentional resources to complex social stimuli.

In line with Senju *et al.* [24], we found a dissociation between implicit and explicit mentalizing tasks in the autism group. Specifically, in the explicit mentalizing task, which is considered an advanced test of mentalizing [50], the performance of autistic adults was indistinguishable from non-autistic adults, indicating sophisticated mentalistic reasoning. On the other hand, in the false-belief condition of our implicit mentalizing task, the non-autism group showed a bias to look at the belief-congruent target location, while the autism group split their time equally between the belief-congruent and -incongruent target locations (i.e. at a chance level), indicating that they did not spontaneously appreciate the agent’s false belief. Of note, this observation could not be accounted for by difficulties predicting actions, submentalizing processes or attentional differences (discussed in more detail below). These findings are consistent with the idea that autistic people without intellectual disability may acquire the capacity to explicitly reason about complex mental states [25], but still struggle to implicitly attribute simple mental states [24].

Despite a marginal general tendency for the autistic group to spend less time looking at the belief-congruent location across conditions, both autistic and non-autistic participants displayed a clear looking bias towards the belief-congruent area in the true-belief conditions, which is consistent with Gliga *et al.*'s [40] familiarization trial results. This deserves detailed consideration as it has distinct implications for the specificity of the mentalizing differences thought to lie at the very core of autism. This finding does not corroborate Heyes’ [35] prediction that autistic people may struggle to spontaneously predict others’ actions *per se*, regardless of mentalizing requirements (also see in Van de Cruys *et al*. [52]), leading to a lack of preference for the belief-congruent location. Furthermore, we also found that both groups of participants showed anticipatory eye movements predicting the agent’s hand reach to retrieve the object in the long familiarization trials (using opaque boxes, based on the first 2500 ms after the onset of the alert signal before the agent reached through the window), implying that both groups were capable of action prediction in this more basic form. These two findings provide evidence against the submentalizing hypothesis and instead indicate good action prediction skills in autism.

Further, in the false-belief and true-belief conditions, both groups of participants spent the majority of their time looking at the puppet rather than the agent’s head while the object was displaced, consistent with a previous study’s analysis of looking behaviour during false-belief trails in infant siblings [40]. Thus, it is unlikely that the non-autistic adults were distracted by the first head-turn and, therefore, failed to notice the critical events (i.e. the object displacement) thereafter, as predicted by the submentalizing hypothesis [35]. Similarly, during the action anticipatory period, the two groups spent a similar amount of time looking at the agent and the object (i.e. windows and boxes), revealing that the autistic adults neither paid more attention to the non-social stimuli nor less attention to the social stimuli than the non-autistic adults, contrary to social attentional theories of autism (e.g. [53]). Likewise, our autistic participants did not show a tendency to fixate on the hidden object location at the end of the videos in the false-belief high-demand condition where the object remained on the scene. This indicates that a reality bias was not preventing them from performing the task any more than for non-autistic adults.

One may argue that the pattern of results could be explained by a last-object-location bias, instead of mentalizing (e.g. [39,54–56]). This alternative strategy predicts belief-congruent performance in the true-belief conditions and the false-belief low-demand condition but belief-incongruent performance in the false-belief high-demand condition. Based on this, both groups’ belief-congruent performance in the true-belief conditions and non-autistic adults’ performance in the false-belief low-demand condition might be achieved by simply looking at the last location the object was hidden without accurately understanding the agent’s belief about the object location. However, the last-object-location bias cannot explain the lack of looking bias in the false-belief high-demand condition in both groups or the reason why autistic adults did not show this bias in the false-belief low-demand condition. Accordingly, this possibility cannot explain, or at least solely explain, the performance of all the conditions in both groups. Indeed, Baillargeon *et al.* [57] commented that the empirical evidence argues against the last-object-location bias in explaining the data pattern from studies using anticipatory-looking paradigms. Therefore, our results cannot be explained by a manifestation of submentalizing, attentional differences, a reality bias or a last-object-location bias. This indicates that autistic adults process information from social cues in the same way as non-autistic adults, but this information may then not be used to update mental representations.

We found a main effect of belief and an interaction between time and belief, neither of which interacted with the group, with participants showing more of a belief-congruent bias in the true- than false-belief condition in the first half of the experiment, although no difference in the second half. While both types of condition involve belief reasoning, it is likely that false-belief reasoning requires more sophisticated mentalizing abilities than true-belief reasoning owing to the need to represent an alternative mental state that differs from one’s own. Indeed, true-belief conditions have consistently performed better than false-belief conditions in the literature [37,38], which indicates that it is easier than false-belief reasoning. Consistent with this, Nijhof *et al.* [58] observed that the right temporoparietal junction was recruited in both true-belief and false-belief reasoning, but more so during false- than true-belief conditions. Similarly, Schneider *et al.* [59] found the same pattern in the superior temporal sulcus. Accordingly, given the differences in performance in our task and differences in brain activation in the literature, false-belief reasoning seems to require a greater degree of mentalizing than true-belief reasoning, although this difference may diminish with exposure. This idea that mentalizing is involved in both true- and false-belief reasoning also helps to explain the marginal main effect of Group, whereby autistic participants showed a tendency to look less at the belief-congruent location across both Belief conditions, despite performing above chance on the true-belief condition but at chance on the false-belief condition.

Considering our false-belief high-demand condition, a looking bias in either group was not observed, consistent with findings from Wang and Leslie [38]. Although this result supports the idea that autistic adults are not affected by a true-belief bias any more than non-autistic adults, it might indicate that this condition failed to elicit mentalizing at all. However, this explanation seems unlikely as the two false-belief scenarios differed only in the final location of the object. A more plausible explanation is that mentalizing test performance, as indexed by anticipatory eye gaze, is also subject to the availability of executive resources, especially inhibitory control [38,60]. Both the true-belief bias hypothesis [38] and the similarity-contingency model [61] propose that people may default to using their own mental states as a basis on which to mentalize others. Taken together with the inhibition model of mentalizing proposed by Leslie and Polizzi [62], it seems likely that an inability to inhibit the specified own belief in the false-belief high-demand condition may have led to greater uncertainty when predicting the agent’s action [38].

Overall, we found that our multi-trial design, to an extent, was sensitive in detecting autistic differences in mentalizing. Increasing the number of trials seemed to effectively decrease not only the high dropout rate seen in previous research but also the error variance, addressing concerns from Dang *et al.* [36] and Kulke *et al.* [33]. To maximize the sensitivity of the task, future research could remove the later blocks and the false-belief high-demand and true-belief short-turn conditions and instead increase the number of false-belief low-demand and true-belief long-turn trials in a single section.

Although we have been able to address some important issues that until now have remained unanswered, an obvious limitation of this study was that all participants were adults and had average-to-high IQs; thus, our findings cannot be generalized to autistic adults with language delay and/or intellectual disability, or autistic children. Still, this non-verbal paradigm holds promise as being adaptable to a much wider range of populations than traditional mentalizing tests. Indeed, future studies should investigate whether spontaneous mentalizing varies between autistic people with different levels of general ability. Additionally, this paradigm, as well as mentalizing in autism more generally, should be further explored, given the main effect of the Group was marginal. Future studies should improve the sensitivity of this paradigm based on the suggestions above to replicate and extend the current study.

## 5. Conclusion

In closing, we extended Southgate *et al.*'s [19] paradigm to critically examine spontaneous mentalizing in autism through a multi-trial, multi-condition eye-tracking study with a more nuanced analysis of eye movements over the timecourse of each trial. Replicating the findings of Senju *et al.* [24], we found that although many autistic people perform well in explicit mentalizing tasks, they do not engage in spontaneous false-belief reasoning in implicit tasks, consistent with their everyday social difficulties. We have been able to rule out alternative theoretical explanations for this pattern of performance, leading to a better understanding of mentalizing in both autistic and non-autistic people. We have presented evidence that autistic adults are capable of processing information from social cues in the same way as non-autistic adults but that this information is not then used to update alternative mental representations. Future studies should directly test the point at which implicit mental state reasoning is hindered in autism, and whether these differences are present throughout the whole autistic population. Non-verbal implicit mentalizing paradigms have the potential to be adapted for use in autism assessments across a wide range of ages and abilities and may prove to be more sensitive and objective than current diagnostic tools, even in the context of camouflaging and to be beneficial for designing personalized support plans.

## Data Availability

The dataset supporting the conclusions of this article is available in the Open Science Framework repository [63]. Supplementary material is available online [64].
